# Oxidative Stress Mediates the Fetal Programming of Hypertension by Glucocorticoids

**DOI:** 10.3390/antiox10040531

**Published:** 2021-03-29

**Authors:** Jeremy Lamothe, Sandhya Khurana, Sujeenthar Tharmalingam, Chad Williamson, Collin J. Byrne, Simon J. Lees, Neelam Khaper, Aseem Kumar, T.C. Tai

**Affiliations:** 1 Biomolecular Sciences, Laurentian University, Sudbury, ON P3E 2C6, Canada; jlamothe@nosm.ca (J.L.); sutharmalingam@nosm.ca (S.T.); nkhaper@nosm.ca (N.K.); akumar@laurentian.ca (A.K.); 2 Medical Science Division, Northern Ontario School of Medicine, Sudbury, ON P3E 2C6, Canada; skhurana@nosm.ca; 3 Chemistry and Biochemistry, Laurentian University, Sudbury, ON P3E 2C6, Canada; 4 Biology, Laurentian University, Sudbury, ON P3E 2C6, Canada; cr_williamson@laurentian.ca (C.W.); cj_byrne@laurentian.ca (C.J.B.); 5 Biology, Lakehead University, Thunder Bay, ON P3E 2C6, Canada; simon.lees@nosm.ca; 6 Medical Science Division, Northern Ontario School of Medicine, Thunder Bay, ON P7B 5E1, Canada

**Keywords:** oxidative stress, glucocorticoids, dexamethasone, fetal programming, antioxidants, epigallocatechin gallate (EGCG), 4-hydroxy-TEMPO (TEMPOL), hypertension, hypothalamic-pituitary-adrenal (HPA) axis, catecholamine biosynthesis, blood pressure

## Abstract

The field of cardiovascular fetal programming has emphasized the importance of the uterine environment on postnatal cardiovascular health. Studies have linked increased fetal glucocorticoid exposure, either from exogenous sources (such as dexamethasone (Dex) injections), or from maternal stress, to the development of adult cardiovascular pathologies. Although the mechanisms are not fully understood, alterations in gene expression driven by altered oxidative stress and epigenetic pathways are implicated in glucocorticoid-mediated cardiovascular programming. Antioxidants, such as the naturally occurring polyphenol epigallocatechin gallate (EGCG), or the superoxide dismutase (SOD) 4-hydroxy-TEMPO (TEMPOL), have shown promise in the prevention of cardiovascular dysfunction and programming. This study investigated maternal antioxidant administration with EGCG or TEMPOL and their ability to attenuate the fetal programming of hypertension via Dex injections in WKY rats. Results from this study indicate that, while Dex-programming increased blood pressure in male and female adult offspring, administration of EGCG or TEMPOL via maternal drinking water attenuated Dex-programmed increases in blood pressure, as well as changes in adrenal mRNA and protein levels of catecholamine biosynthetic enzymes phenylalanine hydroxylase (*PAH*), tyrosine hydroxylase (*TH*), dopamine beta hydroxylase (*DBH*), and phenylethanolamine N-methyltransferase (*PNMT*), in a sex-specific manner. Furthermore, programmed male offspring displayed reduced antioxidant glutathione peroxidase 1 (*Gpx1*) expression, increased superoxide dismutase 1 (*SOD1*) and catalase (*CAT*) expression, and increased pro-oxidant NADPH oxidase activator 1 (Noxa1) expression in the adrenal glands. In addition, prenatal Dex exposure alters expression of epigenetic regulators histone deacetylase (*HDAC*) 1, 5, 6, 7, 11, in male and *HDAC7* in female offspring. These results suggest that glucocorticoids may mediate the fetal programming of hypertension via alteration of epigenetic machinery and oxidative stress pathways.

## 1. Introduction

Hypertension is a key risk factor in the pathogenesis of cardiovascular disease (CVD) which, as of 2016, was the leading cause of death accounting for a total of 15.2 million deaths worldwide [1]. Understanding the mechanisms behind the development of hypertension has remained elusive, although research has highlighted the role of physical activity, sex, ethnicity, heredity, diet, and age. Increasing evidence suggests that the in-utero environment can play a role in post-natal health. Studies show that an adverse fetal environment can program for cardiovascular dysfunction later in life [2]. Fetal adaptation to insults, such as stress, leads to disease; this field known as fetal programming has uncovered a unique origin in the pathology of hypertension, which holds therapeutic potential for prevention of adult disease.

Maternal insults, such as malnutrition or stress (physiological and psychological), can permanently alter tissue structure and function, programming for cardiovascular dysfunction in the fetus [3]. Understanding the effect of the maternal stress response on the fetus is key as ultimately many of these maternal insults lead to elevated stress, increasing the production of the hormone cortisol following hypothalamic-pituitary-adrenal (HPA) axis and sympathetic activation [3]. This maternal stress can be simulated via the maternal administration of exogenous glucocorticoids, such as betamethasone or dexamethasone (Dex) [4], and is often administered to mothers at risk of preterm labor to stimulate fetal lung maturation. Excessive glucocorticoid administration may lead to lifelong health effects, including cardiovascular dysfunction [5].

Administration of Dex throughout the last trimester of pregnancy has been shown to alter the expression of catecholamine biosynthetic enzymes in adult offspring, leading to elevated plasma epinephrine and contributing to hypertensive programming [6,7]. Elevated plasma catecholamines have been linked to individuals with primary hypertension and likely plays a role in pathogenesis [8]. Catecholamine biosynthetic enzymes include: tyrosine hydroxylase (TH), which converts L-Tyrosine to L-Dopa; dopamine beta hydroxylase (DBH), which then forms norepinephrine from Dopamine (a derivative of L-Dopa); and phenylethanolamine N-methyltransferase (PNMT), which converts norepinephrine to epinephrine. Glucocorticoids (GC) bind glucocorticoid receptors (GRs), forming a GR/GC complex which alters gene expression by binding glucocorticoid response elements (GRE) in gene promoter regions, including catecholamine biosynthetic enzymes [3,6]. Recent research has described changes in epigenetic regulators, such as HDACs and DNMTs, which appear to be implicated in programming [7]. Furthermore, a link between these epigenetic regulators and oxidative stress has been highlighted [7].

Reactive oxygen species (ROS) have been shown to impact *PNMT* expression, potentially leading to elevated catecholamines, and glucocorticoids are known to increase ROS [9]. Administration of Dex has been shown to significantly increase ROS production in rat hippocampal slice cultures via interaction with GRs, resulting in increased expression of NADPH oxidase (*Nox*) and reduced expression of glutathione peroxidase (*GPx*) [10]. Research has also linked in utero Dex exposure to elevated superoxide (O_2_^−^) and hydrogen peroxide (H_2_O_2_), leading to cardiovascular programming in sheep [11]. ROS may also be implicated in propagating changes in gene expression into adulthood as ROS can alter epigenetic processes, such as DNA methylation and histone modifications [12].

Research has yet to fully understand the effects of glucocorticoid programming on catecholamine biosynthetic enzyme expression and the protective effect antioxidants may display. Despite strong evidence for a connection, the role of ROS in the glucocorticoid-mediated fetal programming of hypertension is unclear. Some studies have attempted to uncover this connection via the use of maternal antioxidants throughout programming [13]. Dietary antioxidants, such as polyphenols, provide an ideal solution as they are readily available and have displayed cardioprotective properties [14,15]. Furthermore, since glucocorticoids increase O_2_^−^ and H_2_O_2_, it is important to employ an antioxidant that combats these ROS [16]. Polyphenols are ideal as they are potent radical scavengers of hydroxyl radicals, superoxide, and several reactive nitrogen species [17,18]. Furthermore, they can be administered non-invasively, such as supplementation of maternal drinking water, for the prevention of cardiovascular programming [19,20].

In addition to naturally occurring antioxidants, synthetic antioxidants are useful in targeting specific ROS and investigating their impact on fetal programming. 4-hydroxy-TEMPO (TEMPOL) is a membrane permeable, stable, superoxide dismutase (SOD) mimetic that catalyzes the degradation of superoxide radicals [21]. TEMPOL has proven to have some remarkable antihypertensive properties; when administered to hypertensive rats, TEMPOL significantly lowered blood pressure and has been shown to improve nitric oxide sensitivity. [21,22]. In the field of fetal programming, Roghair and colleagues showed that maternal TEMPOL treatment via drinking water attenuated glucocorticoid-mediated cardiovascular programming in mice [13].

Recent data highlights a connection between epigenetics and oxidative stress in the glucocorticoid-mediated fetal programming of hypertension [7]. Specifically, the use of postnatal epigenetic inhibitors, including DNMTi or HDACi, attenuated hypertensive programming and displayed alterations in oxidative stress enzymes, including catalase (*CAT*), NADPH oxidase activator 1 (*Noxa1*), glutathione peroxidase 1 (*Gpx1*), and *SOD* in adrenal tissue [7]. The aim of this research is to unravel the interaction between ROS and glucocorticoids in the fetal programming of hypertension. Specifically, maternal antioxidant administration with epigallocatechin gallate (EGCG) or TEMPOL was employed to determine if ROS are implicated in the glucocorticoid-mediated fetal programming of hypertension.

## 2. Materials and Methods

### 2.1. Animal Protocol 

Animal care procedures were approved by the Animal Care Committee at Laurentian University (AUP2018-02-01), in agreement with the Canadian Council on Animal Care guidelines. Wistar-Kyoto (WKY) rats; male (*n* = 6) and female (*n* = 18), were acquired at 6 weeks old from Charles River Laboratories (Montreal, QC, Canada). Animals were fed standard rat chow from Harlan Tekland (Indianapolis, IN, USA) ad libitum. Further, the light cycle consisted of a 12/12 light/dark period with light between 6:00 a.m. to 6:00 p.m.

### 2.2. Breeding and Experimental Design

Rats were left to acclimate for four weeks until they reached 10 weeks of age. Males were then introduced to females until vaginal plugs were observed. Pregnant females were then singly housed for the remainder of the pregnancy. Following copulation, pregnant rats were randomly assigned (*n* = 6) to specific treatment groups: Control (water), TEMPOL (1 mmol/L; Sigma-Aldrich, St. Louis, MO, USA) [23], or 0.1% EGCG (458 mmol/L; Toronto Research Chemicals, Toronto, ON, Canada). EGCG and TEMPOL were both chosen as they previously demonstrated their cardiovascular protective effects when administered in drinking water; dosing was based on previous studies and is within the range of equivalent human physiological dose [13,24]. EGCG is a common green tea extract, and TEMPOL is an enzyme mimetic and specifically catalyzes the degradation of superoxide. Antioxidant solutions were made fresh daily and were added to drinking water at 6 p.m. each day. 

**Figure 1:**
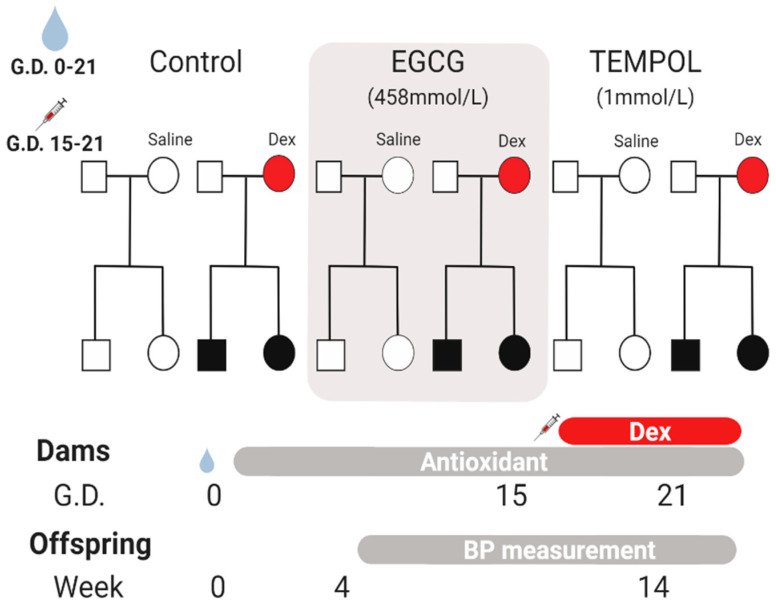
Overview of maternal antioxidant administration and dexamethasone (Dex)-mediated fetal programming. Pregnant WKY dams received antioxidant supplementation or vehicle throughout gestation days 0–21. Fetal programming was achieved using S.C. injection throughout the third trimester, 100 ug/kg days 15–21. Offspring subjected to Dex in-utero are depicted in black. Blood pressure measurements were determined based on a weekly average throughout development from weeks 4–14. Euthanasia occurred at the beginning of week 15, and adrenal glands were harvested for gene expression analysis.

The three previous groups of dams (*n* = 6) (Control, TEMPOL, and EGCG) were then further subdivided to make 6 total groups (*n* = 3). Three dams from each group received a saline vehicle injection (4% ethanol/0.9% saline solution), and the remaining received dexamethasone (0.1 mg/kg per day) sub-cutaneous (S.C.) from gestational day (GD: 15–21) as described previously [6,7]. Offspring from each dam were then randomly chosen within their respective group to form the offspring groups: Control-Saline, Control-Dex, EGCG-Saline, EGCG-Dex, TEMPOL-Saline, TEMPOL-Dex (*n* = 6) (Figure 1).

### 2.3. Blood Pressure

Pups from each group were sexed and weaned at 3 weeks of age and separated into groups of 2–3. Body weight was recorded from week 3 forward to reduce early life handling stress which has been shown to impact HPA-axis, which may skew results [25]. Blood pressure measurements were recorded using the non-invasive volume blood pressure system CODA 8 (Kent Scientific, Torrington, CT, USA) as depicted previously [6,7]. The CODA 8 high throughput system involves the animal being placed into a plexiglass tube and gentle warming on the warming pad provided. Pups were acclimated to restraint and the blood pressure system for a week before measurements (week 3) and for 10 min prior to recording each day’s measurements. Blood pressure was measured thrice a week from weeks 4–14, as previously described [6,7]. Care was taken to ensure measurements were taken prior to any husbandry practices and between 9 a.m. and 6 p.m. to prevent natural diurnal fluctuations in blood pressure.

### 2.4. Tissue Collection and Extraction

At week 14, offspring were anesthetized via intra-peritoneal (I.P.) injection of a solution of 5 mg/kg xylazine (Rompun; Bayer, Etobicoke, ON, Canada) and 75 mg/kg Ketalean (Ketalean; Bimeda, Cambridge, ON, Canada) [26]. Animals were then euthanized using decapitation; trunk blood was collected and put on ice. Tissues, including adrenal glands, were extracted and immediately frozen on dry ice as performed previously [6].

### 2.5. Adrenal mRNA Expression

The left adrenal from each animal was homogenized using the Tissuelyser (Qiagen, Hilden, Germany) with Trizol reagent (Sigma-Aldrich) as previously described [6,7]. Total RNA was extracted, resuspended in DEPC treated nuclease-free water, and RNA concentration determined using a Nanodrop 1000 (Thermo Fisher Scientific, Wilmington, DE, USA) spectrophotometer at an absorbance of 260 nm. To ensure there was no genomic DNA contamination following RNA extraction, samples were treated with the DNase I kit (Sigma-Aldrich). RNA was then converted to cDNA using the reverse transcriptase M-MLV (Promega, Madison, WI, USA) [6]. The expression of catecholamine biosynthetic enzymes and related transcription factors was determined by qPCR (Bioline SensiFast Sybr Lo-Rox mix; CSA-01195 FroggaBio, Concord, ON, Canada), using the Chromo4 qPCR thermocycler (BioRad, Hercules, CA, USA). Primers used have been outline previously [7]. Expression of additional epigenetic and ROS related targets were assessed using a custom RT^2^ profiler array (Qiagen). Samples were run using 7.5 ng input cDNA with a total volume of 15 μL reactions. Primers for *DBH*, *TH*, *PNMT*, specificity protein 1 (*SP1*), early growth response 1 (*EGR-1*), *GR*, *B-Actin*, and *RPL29* were purchased from Sigma-Aldrich. Change in expression was quantified using the Ct value for each sample via the Pfaffl method *ratio* = (*E_target_*) ^*ΔCTtarget(control−sample)*^/(*Eref*) ^*ΔCTref(control−sample)*^ [27]. All samples were analyzed in duplicate, with a total biological sample size of N=6 animals per group unless otherwise indicated.

### 2.6. Western Blot

Western blot was performed as described previously [7]. In summary, protein was extracted from the right adrenal gland of the offspring using the All-Prep kit (Qiagen) [7]. Samples were homogenized using the Tissuelyser (Qiagen) and protocol followed as per manufacturer’s instructions. Dithiothreitol (DTT) was added to the final protein solubilizing buffer (ALO) to a strength of 8 mg DTT/1 mL ALO which aided in dissolution of the protein pellet. Samples were resuspended in 300 μL of ALO buffer, sonicated (10 s at 100% amplitude, Sonic Membrator Model 500 Fisher Scientific, Waltham, MA, USA) to aid in pellet solubilization, then stored at −80 °C until use. Western blot was performed using an input of 5 μL of sample as performed previously [6], and transferred onto PVDF membranes. Blocking was performed using 5% milk, except for the DBH membrane, which was blocked in 2% BSA. Antibodies specific for TH (Novus Biologicals, Littleton, CO, USA) (1:4000), DBH (1:1000), PNMT (1:500), GR (1:1000), GAPDH (Abcam, Cambridge, UK) (1:250,000), and SP1 (Santa-Cruz Biotech, Dallas, TX, USA) (1:250) were incubated overnight at 4 °C. Secondary antibodies conjugated to HRP-IgG (anti-rabbit or anti-mouse) were incubated for 1 h at room temp and used according to primary antibody origin. Membranes were then incubated with enhance chemiluminescence (ECL) for 2 min (as described by Haan and Behrmann 2007) [28] and exposed to a film (CL-XPosure Film 34091, Thermo Scientific). Quantification of protein bands was assessed with ImageJ (U.S. National Institutes of Health, Bethesda, MD, USA). All protein quantified were normalized to GAPDH. Western blot for EGR1 and phenylalanine hydroxylase (PAH) was not performed due to the lack of suitable primary antibodies for analysis.

### 2.7. Corticosterone and Catecholamine Levels

Blood was stored in Vacutainer blood collection vials with EDTA (Becton Dickinson, Franklin Lakes, NJ, USA) and centrifuged at 1500× *g* for 20 min as performed previously [6]. Plasma was then isolated and frozen at −80 °C until use. Epinephrine and norepinephrine levels were quantified using the 2-CAT Labour Diagnostika Nord (LDN) (Rocky Mountain Diagnostic, Colorado Springs, CO, USA) ELISA. For each sample, 50 μL of plasma was thawed on ice and used in the ELISA as per manufacturer’s instructions. The Parameter corticosterone ELISA was employed to assess plasma corticosterone levels from R&D Systems (Minneapolis, MN, USA).

### 2.8. Quantification and Statistical Analysis

GraphPad PRISM software (La Jolla, CA, USA) was employed to assess statistical significance via two-way ANOVA (Fisher’s LSD test) factoring for maternal injections and or antioxidant administration with a *p* ≤ 0.05 reaching significance. Significant interactions were detected and have been included in figure legends. All data is presented as mean ± SEM.

## 3. Results

### 3.1. Physiological Measurements

**Figure 2:**
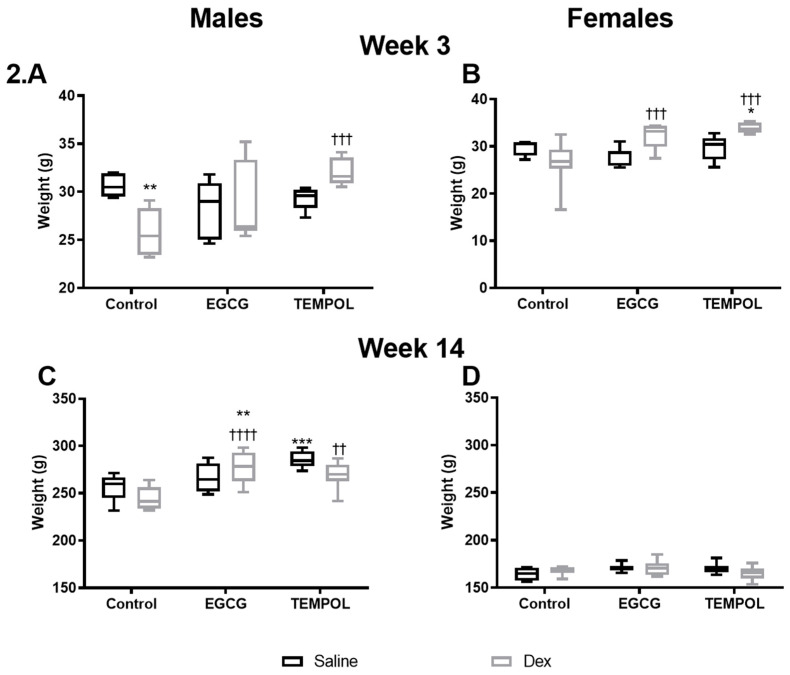
Offspring body weight. The effects of maternal antioxidant administration on offspring body weight at week 3 for males (**A**) and females (**B**) and week 14 males (**C**) and females (**D**) in programmed (Grey) and unprogrammed animals (Black). Saline and Dex control have been published previously and have been provided for reference [7]. Two-way ANOVAs were performed (Fisher’s LSD test). * is used to indicate significance; * *p* ≤ 0.05, **/†† *p* ≤ 0.01, ***/††† *p* ≤ 0.001, †††† *p* ≤ 0.0001. Comparison to the Control-Saline group is represented with the symbol (*) and (†) for the Control-Dex offspring. N = 6 per group. Significant interactions were present for week 3 and 14 between maternal injection and antioxidant administration for males and females.

**Figure 3:**
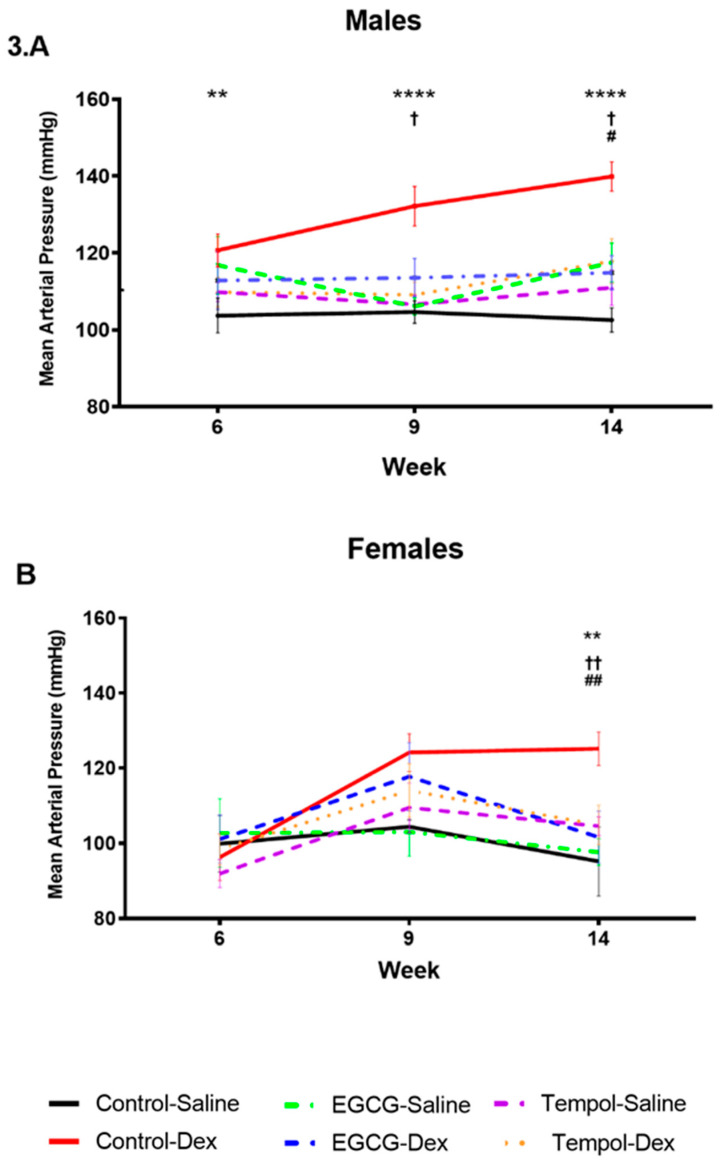
The impact of in-utero antioxidant administration on mean arterial pressure in Dex programmed offspring. Mean arterial pressure of Dex-exposed animals administered EGCG or TEMPOL in-utero throughout gestation in males (**A**) and females (**B**). Measured using the CODA 8 blood pressure monitor from Kent Scientific. Saline and Dex control have been published previously and have been provided for reference [7]. Two-way ANOVA (Fisher’s LSD test): significance is shown as †/# *p* ≤ 0.1, **/††/## *p* ≤ 0.05, **** *p* ≤ 0.0001. The symbols (*/†/#) represent statistical significance; (*) between Control-Saline and Control-Dex, (†) between Control-Dex and Tempol-Dex and (#) between Control-Dex and epigallocatechin gallate (EGCG)-Dex. *n* = 6 per group. Significant interactions between maternal injection and antioxidant administration were found at week 14 for males and females.

As reported previously [7], Dex-exposed males displayed decreased post-natal weight at 3 weeks compared to saline control. (Figure 2A). Maternal supplementation with the antioxidant EGCG had no effect on postnatal weight compared to saline control; however, it did impact rats prenatally exposed to Dex, recovering body weight comparable to saline control animals (Figure 2A,C). In contrast, maternal TEMPOL supplementation in the saline group increased post-natal weight of males at 14 weeks, and attenuated Dex-mediated reductions in body weight at week 3 (Figure 2A,C). In Dex-exposed females, TEMPOL increased weight at week 3 compared to both saline and Dex control groups (Figure 2B). Prenatal Dex exposure increased mean arterial pressure (MAP) in both male (140 mmHg) and female (125 mmHg) rats at 14 weeks of age compared to saline (103 mmHg and 95 mmHg, respectively) (Figure 3A,B), as described previously [7]. Maternal supplementation with EGCG or TEMPOL significantly attenuated prenatal Dex-induced increases in blood pressure in both males and females by week 14 (Figure 3A,B).

### 3.2. Transcript Analysis 

**Figure 4:**
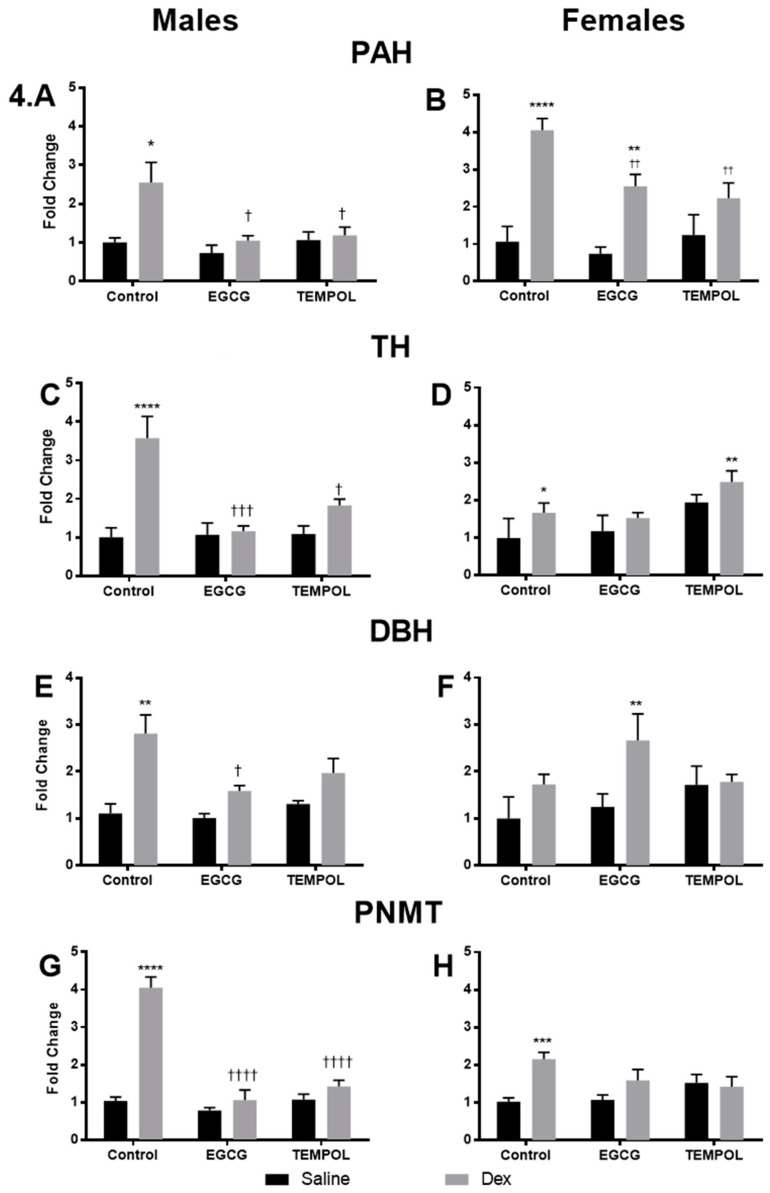
The impact of in-utero antioxidant administration on adrenal catecholamine biosynthetic enzyme expression levels. Quantification of adrenal catecholamine biosynthetic enzyme levels was performed using qPCR and included: phenylalanine hydroxylase (*PAH*) (**A**,**B**), tyrosine hydroxylase (*TH*) (**C**,**D**), dopamine beta hydroxylase (*DBH*) (**E**,**F**), and phenylethanolamine N-methyltransferase (*PNMT*) (**G**,**H**) for males and females, respectively. Saline and Dex control have been published previously and have been provided for reference [7]. Two-way ANOVA (Fisher’s LSD test): significance is shown as */† *p* ≤ 0.05, **/†† *p* ≤ 0.01, ***/††† *p* ≤ 0.001, ****/†††† *p* ≤ 0.0001. Comparison to the Control-Saline group is represented with the symbol (*) and (†) for the Control-Dex offspring. N = 4–6 per group. Data are depicted as mean ± SEM. Significant interactions between maternal injection and antioxidant administration were found for PAH and PNMT males and females, as well as TH males.

Saline and Dex control data has been previously reported with the exception of PAH and is provided for reference [7]. Male Dex-exposed offspring displayed increases in the expression of PAH (2.5-fold) compared to control (Figure 4A). Previous data has shown similar trends in the expression of TH, DBH, PNMT genes in the Dex-exposed offspring compared to the Control-Saline group (Figure 4C,E,G) [7]. Female Control-Dex offspring also displayed similar trends with an increase in PAH (4.1-fold) compared to saline control (Figure 4B). Once again similar but less robust changes in the expression of TH and PNMT (Figure 4B,D) were observed, which have been described previously [7].

EGCG alone had no effect on gene expression in unprogrammed male or female offspring. However, EGCG attenuated Dex-mediated increases in PAH (1.1-fold), TH (1.2-fold), DBH (1.6-fold), and PNMT (1.1-fold) in males (Figure 4A–H). This is in contrast to Dex-exposed females, which only displayed a slight attenuation in TH expression (1.5-fold) (Figure 4D). Females in the EGCG-Dex group also showed a reduction in PAH expression from Control-Dex (2.6-fold from 4.1-fold) offspring, which remains significantly elevated compared to control (Figure 4B). In contrast to males, EGCG administration resulted in further increases in DBH expression in Dex-exposed females (2.7-fold), more than even Control-Dex (1.7-fold) animals (Figure 4F).

Administration of TEMPOL alone did not alter the expression of catecholamine biosynthetic enzymes in males; however, interestingly, females displayed increased TH expression (1.9-fold) compared to Control-Saline (Figure 4D). Similar to EGCG administration, TEMPOL attenuated Dex-programmed increases in catecholamine related enzymes; PAH (1.2-fold), TH (1.8-fold), DBH (2.0-fold), and PNMT (1.4-fold); however, DBH and TH remain elevated compared to control in males (Figure 4A,C,E,G). Dex-exposed females, however, present more complicated catecholamine producing enzyme expression patterns when given TEMPOL. Similar to EGCG, TEMPOL diminished Dex-mediated increases in female PAH expression (2.2-fold from 4.1); however, it remained elevated compared to control (Figure 4B). In contrast to males, TEMPOL was not successful in attenuating increased TH expression in Dex-programmed females, which is increased further from Control-Dex (2.5-fold from 1.7) (Figure 4D). Similar to males, TEMPOL was effective in preventing increased PNMT expression in Dex-exposed females compared to the Control-Dex group (down to 1.5 from 2.2-fold) (Figure 4H).

**Figure 5:**
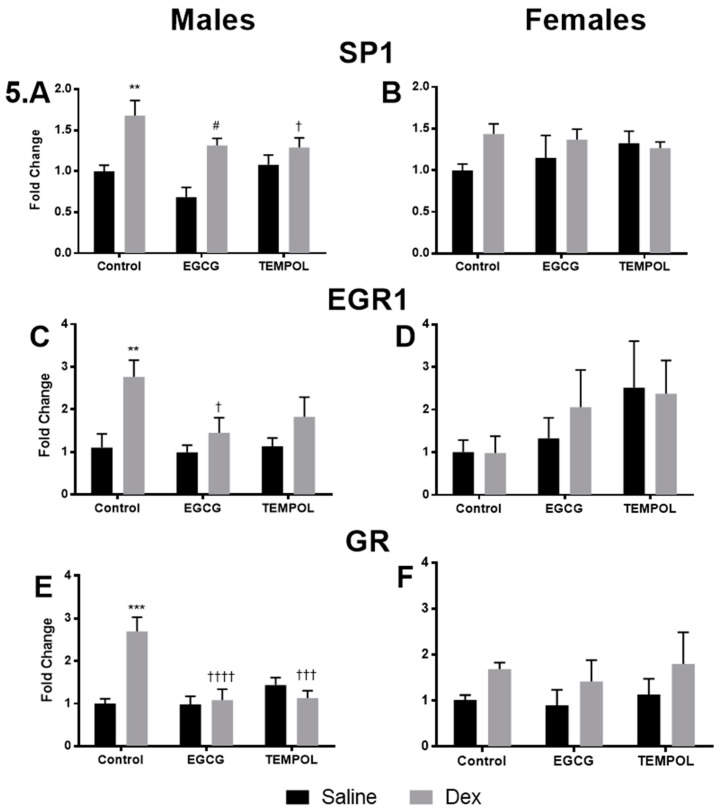
Relative mRNA expression of transcription factors involved in catecholamine biosynthetic enzyme expression (2 ^−ΔΔCt^). qPCR gene expression of transcription factors specificity protein 1 (*SP1*) (**A**,**B**), early growth response 1 (*EGR1*) (**C**,**D**), and glucocorticoid receptor (*GR*) (**E**,**F**) for males and females, respectively. Saline and Dex control have been published previously and have been provided for reference [7]. Results from a Two-way ANOVA (Fisher’s LSD test) are shown, significance is depicted as † *p* ≤ 0.05, ** *p* ≤ 0.01, ***/††† *p* ≤ 0.001. Comparison to the Control-Saline group is represented with the symbol (*), (†) for the Control-Dex offspring and (#) is relative to the EGCG-Saline group. *n* = 4–6 per group. Data are presented as mean ± SEM. Significant interactions between maternal injection and antioxidant administration were found for GR males.

Saline and Dex control transcription factor data has been previously reported and has been provided for reference [7]. Prenatally Dex-exposed male offspring also displayed altered expression of transcription factors, including increased expression of SP1, EGR1, and GR (Figure 5A,C,E) [7]. Dex-exposed females display similar trends in the expression of SP1 and GR, but not EGR1 compared to control (Figure 5B,D,F). EGCG alone did not impact the expression of transcription factor in either sex (Figure 5A–F). However, EGCG was successful in preventing Dex-mediated increases in SP1 (1.3-fold), EGR1 (1.5-fold), and GR (1.1-fold) (Figure 5A,C,E) in males but not females. EGCG-Dex females displayed trends in elevated SP1 (1.4-fold) and GR (1.7-fold) expression compared to control although not significant (Figure 5B,D).

TEMPOL alone did not significantly alter the expression of any transcription factors for either sex. Furthermore, similar to EGCG, TEMPOL attenuated the increased expression of SP1 (1.3), EGR1 (1.8-fold), and GR (1.1-fold) in Dex-exposed male offspring and expression levels are comparable to control groups (Figure 5A,C,E). In Dex-exposed females, TEMPOL has trended for reduced SP1 expression (1.3-fold) (Figure 5A). However, EGR1 (2.4-fold) and GR (1.8-fold) maintain elevated trends in expression that have not reached significance over control animals (Figure 5C,E).

### 3.3. Protein Analysis

**Figure 6:**
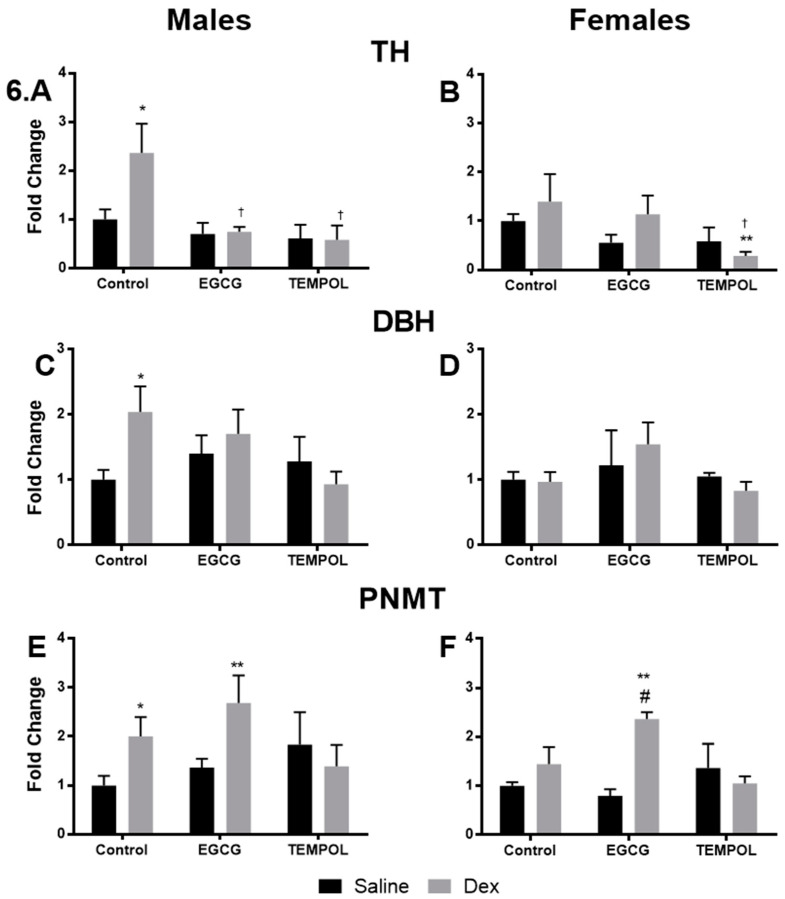
The impact of–in-utero antioxidant administration on adrenal catecholamine biosynthetic enzyme protein levels. Results of western blot for male and female tyrosine hydroxylase (TH) (**A**,**B**), dopamine beta hydroxylase (DBH) (**C**,**D**), phenylethanolamine N-methyltransferase (PNMT) (**E**,**F**) exposed to TEMPOL or EGCG in utero. Saline and Dex control have been published previously and have been provided for reference [7]. Statistical analysis was performed using a two-way ANOVA (Fisher’s LSD test) shown as */†/# *p* ≤ 0.05, ** *p* ≤ 0.01. Data are presented as mean ± SEM. Comparison to the Control-Saline group is represented with the symbol (*), (†) for the Control-Dex offspring and (#) relative to EGCG-Saline. N = 4–6 per group. Significant interactions between maternal injection and antioxidant administration were found for PNMT females.

Saline and Dex control data has been previously reported and is provided for reference [7]. The in-utero exposure of DEX resulted in increased catecholamine biosynthetic enzyme protein levels for TH, DBH, and PNMT in males, however, not in female offspring (Figure 6A,C,E), although females in the Control-Dex cohort do show a trend for increasing expression of TH and PNMT (Figure 6B,F).

EGCG alone did not significantly alter protein level for catecholamine enzymes in males or females. Interestingly, EGCG attenuated elevated levels of TH (0.7-fold) and DBH (1.7-fold) but not PNMT (2.7-fold) in Dex-exposed males (Figure 6A,C,E). Like males, EGCG-Dex females displayed increased levels of PNMT (2.4-fold) compared to control (Figure 6F).

TEMPOL alone did not result in increased protein levels of catecholamine enzymes. Like EGCG, TEMPOL prevented Dex-mediated increase in TH (0.6-fold) and DBH (0.9-fold) but also PNMT (1.4-fold) in males (Figure 6A,C,E). In females exposed to Dex, a significant reduction in TH levels (0.3-fold) were seen with TEMPOL (Figure 6B).

**Figure 7:**
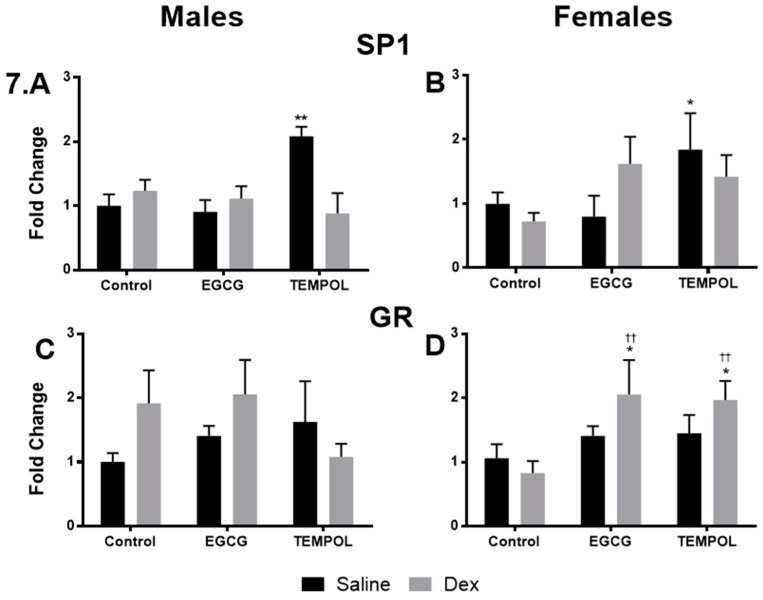
Quantification of transcription factor protein level from offspring adrenal gland. Results of western blot for male and female specificity protein 1 (SP1) (**A**,**B**) and glucocorticoid receptor (GR) (**C**,**D**) exposed to TEMPOL or EGCG in utero. Saline and Dex control have been published previously and have been provided for reference [7]. Statistical analysis was performed using a two-way ANOVA (Fisher’s LSD test) shown as * *p* ≤ 0.05, **/†† *p* ≤ 0.01. Data are presented as mean ± SEM. Comparison to the Control-Saline group is represented with the symbol (*) and (†) for the Control-Dex offspring *n* = 4–6 per group. Significant interactions between maternal injection and antioxidant administration were found for SP1 males.

Once again saline and Dex control data has been provided for reference [7]. Interestingly Dex-exposure did not lead to increased protein levels in either sex despite elevations in mRNA expression levels (Figure 7A,B). Dex-exposed male offspring do display a trend for increased GR levels (1.7-fold) nearing significance; however, this is not seen in females (Figure 7C). No significant results were found between EGCG-Dex and control in male offspring; however, EGCG-Dex males display a trend towards increased GR (2.1-fold) (Figure 7C). In contrast, EGCG in combination with Dex resulted in a trend for increased SP1 (1.6-fold) and significantly increased (2.1-fold) GR in females (Figure 7B,D).compared to control.

Once again contrary to gene expression data, TEMPOL alone significantly increased SP1 levels in males (2.1-fold) and females (1.8-fold) but no change in GR (Figure 7A–D). In combination with Dex, TEMPOL administration resulted in a trend in GR levels comparable to control in males (1.1-fold) and increased in females (2.0-fold) (Figure 7C,D).

### 3.4. Plasma Corticosterone and Catecholamines

**Figure 8:**
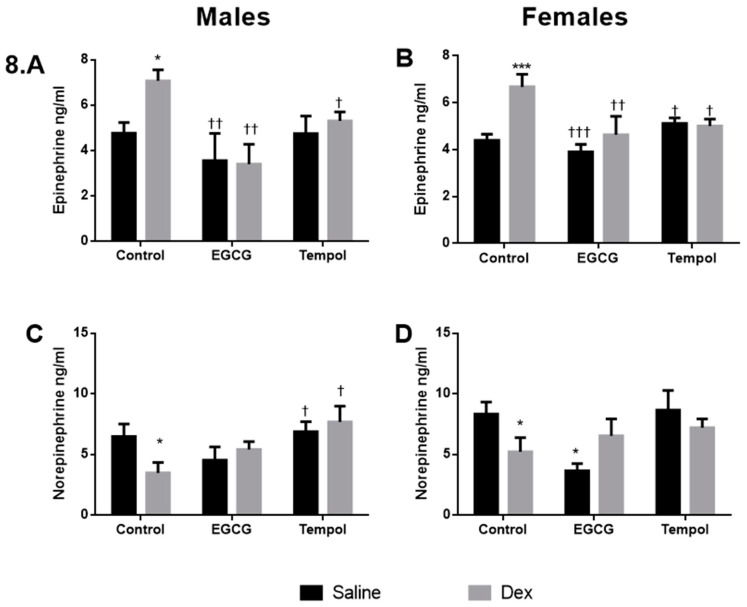
Plasma Catecholamine levels. Results from plasma catecholamine ELISA for epinephrine (**A**,**B**) and norepinephrine (**C**,**D**) for males and females, respectively. Saline and Dex control have been published previously and have been provided for reference Statistical analysis was performed using a two-way ANOVA (Fisher’s LSD test) shown as */† *p* ≤ 0.05, †† *p* ≤ 0.01, ***/††† *p* ≤ 0.001. Data are presented as mean ± SEM. Comparison to the Control-Saline group is represented with the symbol (*) and (†) for the Control-Dex offspring *n* = 4–6 per group. Significant interactions between maternal injection and antioxidant administration were found for epinephrine females.

Plasma catecholamine levels were quantified via ELISA as discussed previously, there were no significant changes in corticosterone levels between groups. [7]. EGCG alone did not alter epinephrine levels in either sex. However, EGCG attenuated increases in epinephrine for Dex-exposed males (3.4 ng/mL) and females (4.6 ng/mL) (Figure 8A,B). Like EGCG, TEMPOL alone had no effect on epinephrine levels for either sex and was successful in attenuating Dex-mediated increases in epinephrine levels for males (5.3 ng/mL) and females (5.0 ng/mL) (Figure 8A,B).

Previous results have shown that in contrast to epinephrine levels, there is a reduction in norepinephrine levels in response to prenatal Dex-exposure in males and females [7]. EGCG alone did not affect norepinephrine levels in male offspring; however, it did significantly reduce levels (3.7 ng/mL) in females compared to control (8.3 ng mL), which was comparable to Control-Dex (5.2 ng/mL) (Figure 8D). EGCG maintained norepinephrine levels in males (5.4 ng/mL) and females (6.5 ng/mL) exposed to Dex compared to Control-Dex offspring (Figure 8C,D). Finally, TEMPOL alone did not alter norepinephrine levels for either sex, and when combined with Dex did not decrease norepinephrine levels in males or females compared to saline control (Figure 8C,D).

### 3.5. RT^2^ Profiller Array

**Figure 9:**
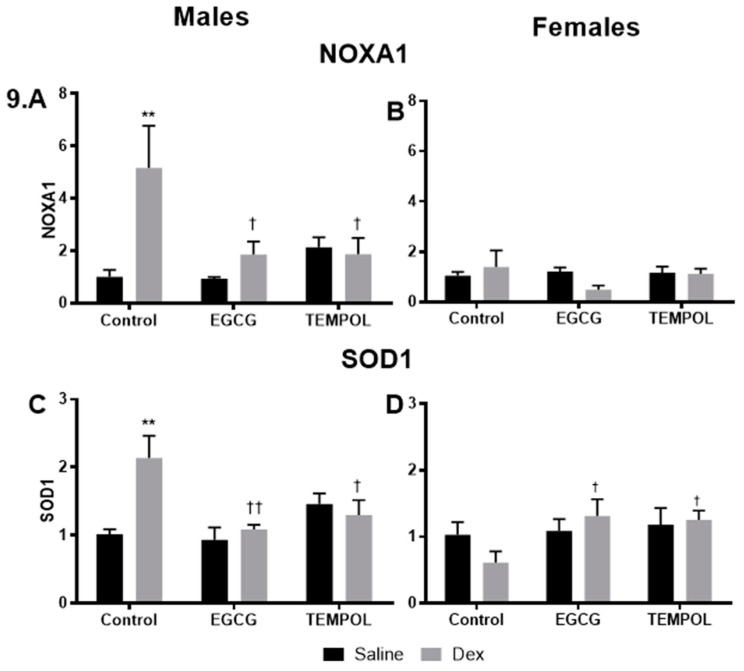
NOXA1 and SOD1 gene expression results from the RT^2^ profiler array (Qiagen). Results from the RT^2^ profiler array for NADPH oxidase activator 1 (NOXA1) (**A**,**B**) and superoxide dismutase 1 (SOD1) (**C**,**D**) for males and females, respectively. Saline and Dex control have been published previously and have been provided for reference [7]. Statistical analysis was performed using a two-way ANOVA (Fisher’s LSD test) shown as † *p* ≤ 0.05, **/†† *p* ≤ 0.01. Data are presented as mean ± SEM. Comparison to the Control-Saline group is represented with the symbol (*) and (†) for the Control-Dex offspring. *n* = 3–5 per group. Significant interactions between maternal injection and antioxidant administration were found for SOD1 males.

As shown previously Dex-exposed males display increased levels of CAT, GPx1, NOXA1, and SOD1 [7]. Interestingly, EGCG and TEMPOL restore expression levels of NOXA1 (1.8 and 2.-fold) and SOD1 (1.1 and 1.3-fold) compared to Control-Saline in male offspring (Figure 9A,C). Dex-exposed females display significantly increased expression of SOD1 when combined with EGCG (1.3-fold) or TEMPOL (1.3-fold) compared to Dex alone (0.6) but were not statistically different then Control-Saline (Figure 9D).

**Figure 10:**
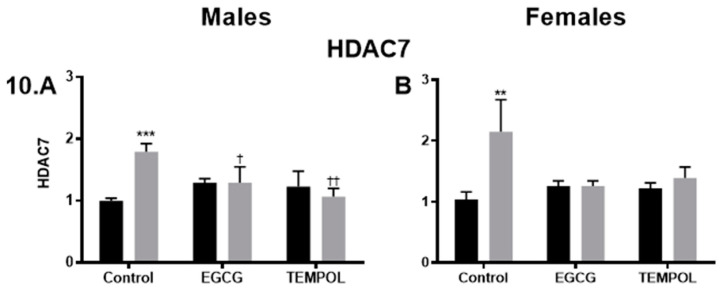
HDAC7 gene expression results from the RT^2^ profiler array (Qiagen). Histone deacetylase (HDAC) 7 (**A**,**B**) gene expression results for males and females, respectively. Saline and Dex control have been published previously and have been provided for reference [7]. Statistical analysis was performed using a two-way ANOVA (Fisher’s LSD test) shown as † *p* = 0.05, **/†† *p* = 0.01, *** *p* = 0.001. Data are presented as mean ± SEM. Comparison to the Control-Saline group is represented with the symbol (*) and (†) for the Control-Dex offspring. *n* = 3–5 per group. Significant interactions between maternal injection and antioxidant administration were found for HDAC7 males.

As discussed previously, epigenetic regulators HDACs (1, 5, 6, 7, 11) displayed increased expression in Dex-exposed males, with females reaching significance in HDAC7 alone [7]. Both EGCG and TEMPOL attenuated Dex-mediated increases in HDAC7 in males (1.3- and 1.1-fold) with females showing similar trends (1.3- and 1.4-fold) (Figure 10B,D). Similar trends in attenuated expression when administered EGCG or TEMPOL can be seen in HDAC1 6 and 11, although not as robust and did not reach statistical significance (data not shown). 

## 4. Discussion

Prenatal exposure to Dex has previously been shown to reduce birth weight and result in increased blood pressure, mRNA and protein levels of catecholamine biosynthetic enzyme levels, as well as circulating levels of epinephrine [6]. Previous research has highlighted altered levels of epigenetic machinery which are likely implicated in these programming effects [7]. Amongst increases in the expression of epigenetic regulators, such as *HDACs 1*, *5*, *6*, *7*, *11*, evidence for alterations in ROS production and antioxidant pathways were discovered, which include *CAT*, *Gpx1*, *Noxa1*, and *SOD1* [7]. Therefore, it is likely that both epigenetics and ROS are implicated in the fetal programming of hypertension via glucocorticoids. The exact mechanism by which ROS contribute to programming remains unknown [13,29]. Von Bergen and colleagues describe Dex-mediated increases in mitochondrial H_2_O_2_ production from complex I in programmed sheep [29]. Interestingly these animals show increased catalase activity in an attempt to compensate for increased hydrogen peroxide production [29]. Dex has also been shown to reduce *GPx* mRNA and increase *Nox* expression in vitro, further highlighting a role for superoxide in programming [10,16]. Glucocorticoids also lead to elevated ROS and reactive nitrogen species through non-genomic regulation of inducible nitric oxide synthase (iNOS) [30]. Once elevated, ROS alter catecholamine producing enzyme production, including PNMT, likely driving increased catecholamine production [9].

Results from the current study show that maternal administration of antioxidants, EGCG or TEMPOL, significantly attenuate the development of the hypertensive phenotype, highlighting the role of ROS in glucocorticoid-mediated fetal programming. Specifically, antioxidants were effective particularly in males, in preventing decreased bodyweight at week 3, elevations in blood pressure, and the majority of altered enzyme expression and protein levels, as well as restoring plasma catecholamine levels in response to prenatal Dex-exposure. 

### 4.1. EGCG and TEMPOL Attenuate Programmed Hypertensive Phenotype

Low birth weight is indicative of Dex-mediated fetal programming of hypertension [6,31]. Antioxidants, such as EGCG, have shown much promise in the treatment and prevention of cardiovascular dysfunction [15,32,33]. Administration of TEMPOL or EGCG during pregnancy recovered offspring weight at week 3, indicating a protective effect of these maternal antioxidants on programming (Figure 2A,B). Both antioxidants in combination with Dex-exposure displayed increased bodyweight over Dex control, and in combination with prenatal Dex exposure EGCG increased weight at 14 weeks compared to control males (Figure 2C). This was also observed in un-exposed TEMPOL males (Figure 2C). Similar results are shown with respect to blood pressure, as animals given either antioxidant in utero display blood pressure levels significantly reduced from Control-Dex offspring (Figure 3A,B). Similar findings by Roghair and colleagues (2011) have shown carbenoxolone programmed mice displayed increased aortic reactivity, which was attenuated with TEMPOL [13].

### 4.2. The Impact of Oxidative Stress on Catecholamine Biosynthesis in Dex-Exposed Offspring

Overall gene expression results suggest a significant attenuation of catecholamine biosynthetic enzymes in response to Dex with maternal antioxidant administration. Maternal consumption of EGCG or TEMPOL resulted in significant decreases in the expression of *PAH*, *TH*, *DBH*, and *PNMT* (Figure 4A,C,E,G) and transcription factors *SP1*, *EGR1*, and *GR* (Figure 5A,C,E) in male programmed offspring. In males, protein levels match mRNA trends apart from PNMT and GR in offspring exposed to EGCG (Figure 6E and Figure 7C).

In contrast to males, programmed females exposed to EGCG or TEMPOL show a reduction, but not complete attenuation, in some catecholamine biosynthetic enzyme expression, including *PAH* and *PNMT* (Figure 4B,H). Furthermore, in some instances, antioxidant in combination with Dex further increased enzyme expression as seen with EGCG and *DBH* (Figure 4F) and TEMPOL and *TH* (Figure 4D). Interestingly, EGCG appears to have exaggerated the effect of programming on PNMT protein levels for both males and females. In general, antioxidant administration prevented increases in catecholamine biosynthetic enzymes with some exceptions. EGCG-mediated increases in PNMT protein do not correlate with elevated blood pressure and the development of hypertension, likely due to regulation of enzymes upstream of PNMT in catecholamine biosynthesis, providing less substrate for catalysis.

Our study provides strong evidence that ROS plays a significant role in programming as males display altered expression of the enzymes involved in the redox pathway, which was remediated with antioxidant administration. It is difficult to elucidate the mechanism by which EGCG may mediate this effect as EGCG can alter transcription, translation and proteasomal degradation of proteins [34,35]. Furthermore, whether antioxidant administration impacts fetal ROS, maternal ROS or both is unknown in the context of fetal programming. EGCG has been shown to pass the placental barrier and accumulate in fetal tissue [36]. EGCG is able to bind RNA, DNA, and protein and has been used as a tool for increasing the effectiveness of RNAi therapies [37]. Binding of EGCG with siRNA prevents its degradation via RNase [37]. As factors regulating PNMT via RNAi and its degradation are unknown, EGCG may be implicated in one or both pathways.

TEMPOL was particularly effective in attenuating Dex programming, providing further evidence that fetal Dex-exposure drives oxidative stress via superoxide generation as TEMPOL was able to prevent these shifts. In females, TEMPOL had less of an effect on reducing catecholamine producing enzyme expression, especially *TH*, suggesting that females display different ROS generation or antioxidant capacity compared to males (Figure 4D,F). This may explain sex-specific differences in programming and response to antioxidant administration. A similar programming study involving prenatally CBX-exposed in mice showed that maternal TEMPOL administration was effective in attenuating increased aortic-reactivity in males and not females [13]. Indeed, there is evidence for altered expression of *Nox* (a predominant generator of superoxide which is catabolized by TEMPOL) between sexes, and has been implicated in the pathogenesis of hypertension [38,39]. Sex specific differences in ROS generation in the heart and brain have been identified previously [40]. Thus, it is plausible that potential differences in ROS generation and antioxidant capacity in the adrenal gland between sexes impact ROS generation and consequently gene expression as a result of Dex-mediated programming. There is evidence that the lower ROS production and greater antioxidant potential present in females is due in part to the protective effects of estrogen [41]. This may play a role in fetal programming as female offspring have been shown to be less susceptible to programming by Dex [42]. The role of estrogen and the estrogen receptor in the fetal programming of cardiovascular dysfunction has been discussed in detail previously, suggesting their role in multiple pathways, such as affecting ROS-mediated catecholamine enzyme programming, increasing vasodilation through eNOS production, and affecting RAS gene expression through DNA methylation [43]. Estradiol has been shown to alter *GR* expression, and there is a complex interplay between *GR* and estrogen receptor in promoting gene expression at estrogen response elements in gene promoter regions, further complicating the mechanism behind estrogen’s protective effects in glucocorticoid programming [44].

Dex-programming has been shown to lead to elevated plasma epinephrine in adult offspring as a result of increased catecholamine biosynthetic enzymes [6]. Indeed, results from the catecholamine ELISA show elevated epinephrine in programmed offspring (Figure 8A,B) and decreased norepinephrine (Figure 8C,D) for both sexes. Maternal antioxidant administration of EGCG or TEMPOL attenuated the programming effects of Dex on offspring plasma catecholamine levels. This was expected of TEMPOL as TEMPOL displayed decreased PNMT expression and protein levels (Figure 4G,H and Figure 6E,F), suggesting less available PNMT for production of epinephrine. However, EGCG displays elevated PNMT protein levels in programmed offspring despite decreased mRNA expression levels (Figure 4G,H and Figure 6E,F). Interestingly EGCG shows a decrease in overall epinephrine levels in plasma of programmed offspring despite elevated PNMT (Figure 8A,B). Previous studies have suggested EGCG lowers circulating catecholamines in response to caffeine; however, the mechanism is unknown particularly within the context of fetal programming [45]. Other factors may be responsible for this, including a reduction in upstream enzymes, as mentioned above, alteration of PNMT catalytic activity, and a shift in the rate of monoamine oxidase activity in the plasma.

Overall, both antioxidants were effective in reducing circulating catecholamine levels, likely a result of significant attenuations in altered catecholamine enzyme levels. Further, antioxidants prevent Dex-mediated changes in these enzymes differently based on sex, indicating ROS differences based on sex in the programming of adrenal catecholamine enzymes.

### 4.3. Additional Mechanisms in Glucocorticoid Programming; ROS and Epigenetics

A qPCR gene mini array was employed to provide insight into the mechanism involved in maternal antioxidant administration during programming, genes targets were selected from ROS/antioxidant, as well as epigenetic pathways, as discussed previously [7]. The results from the analysis of RT^2^ qPCR data indicate that significant changes occur in antioxidant defense enzymes, as well as epigenetic regulators in programmed male offspring, compared to unprogrammed (Figure 9A,C and Figure 10A). Of note, programmed males show a robust increase in NADPH oxidase activator 1 (*Noxa1*) (Figure 9A) further supporting sex-specific increases in factors involved in superoxide generation [7]. Interestingly, both EGCG and TEMPOL significantly reduced Noxa1 expression in programmed male offspring (Figure 9A). Other studies demonstrate increased mitochondrial superoxide and hydrogen peroxide in Dex programmed offspring [11]. Our study provides evidence that males may attempt to compensate for elevations in ROS via an increase in *SOD1* expression (Figure 9C), which is not seen in the females. As shown previously, *GPx1* expression is significantly decreased in programmed male offspring [7], which likely contributes to excessive hydrogen peroxide buildup. Furthermore, there appears to be a compensatory increase in *CAT* in Dex programmed rats and sheep [7,29], which reduces hydrogen peroxide to form water and oxygen.

As described previously, programming has increased the expression of epigenetic regulators, including *HDACs 1*, *5*, *6*, *7*, and *11* in males, may contribute to ROS generation and altered antioxidant defense enzymes, leading to programming. Females, on the other hand, displayed only increased *HDAC7* and seemed to be protected from altered antioxidant enzyme expression, likely contributing to less programming of catecholamine production [7].

There are likely numerous links between oxidative stress enzymes and epigenetic regulators which have remained undiscovered. However, research has described increased HDACs driving increased *Nox* levels, leading to increased superoxide production [46]. Furthermore, HDAC inhibition has been shown to inhibit Nox subunit expression and attenuate the development of hypertension present in the spontaneous hypertensive rat (SHR) [47]. Indeed, previous findings have demonstrated that Dex-programmed offspring given HDAC inhibitors in adulthood remediate changes in altered antioxidant defense enzymes, including *CAT*, *NOXA1*, and *SOD1* [7].

## 5. Conclusions

It is evident that many factors are implicated in the development of cardiovascular disease. The fetal environment is extremely fragile and susceptible to insult from excess glucocorticoids due to maternal stress. Increasing evidence points to epigenetics and ROS in the glucocorticoid programming of cardiovascular dysfunction. Our findings support studies that show maternal antioxidant therapy is a useful tool in the prevention of cardiovascular programming [13]. Many antioxidants researched previously for their use post-natally are being re-evaluated for their potential to attenuate fetal cardiovascular dysfunction. Our study demonstrates that administration of TEMPOL or EGCG attenuated the hypertensive phenotype due to Dex programming in both sexes. In response to prenatal Dex-exposure, male and female programmed offspring displayed altered expression of catecholamine biosynthetic enzyme levels (*PAH*, *TH*, *DBH*, *PNMT*). However, compared to females, males disproportionately showed severe dysregulation of the antioxidant defense enzyme (*SOD1*), increased expression of pro-oxidant activator gene (*Noxa1*) and altered HDAC 7 expression levels. Altered epigenetic regulators, such as HDACs, have been implicated in glucocorticoid programming, and there is evidence they alter expression levels of enzymes in the antioxidant pathway, increasing ROS, and affecting glucocorticoid programming [7]. When combined with our previous study, this research solidifies a relationship between oxidative stress and epigenetics in programming, particularly in males [7]. Further, maternal antioxidant administration via drinking water is an effective strategy in preventing hypertensive programming due to glucocorticoid driven dysregulation of oxidative stress and epigenetic machinery pathways.

## Data Availability

All relevant data are within the manuscript files.
